# Baselines and Degradation of Coral Reefs in the Northern Line Islands

**DOI:** 10.1371/journal.pone.0001548

**Published:** 2008-02-27

**Authors:** Stuart A. Sandin, Jennifer E. Smith, Edward E. DeMartini, Elizabeth A. Dinsdale, Simon D. Donner, Alan M. Friedlander, Talina Konotchick, Machel Malay, James E. Maragos, David Obura, Olga Pantos, Gustav Paulay, Morgan Richie, Forest Rohwer, Robert E. Schroeder, Sheila Walsh, Jeremy B. C. Jackson, Nancy Knowlton, Enric Sala

**Affiliations:** 1 Center for Marine Biodiversity and Conservation, Scripps Institution of Oceanography, La Jolla, California, United States of America; 2 National Center for Ecological Analysis and Synthesis, University of California Santa Barbara, Santa Barbara, California, United States of America; 3 National Oceanic and Atmospheric Administration (NOAA) Fisheries Service, Pacific Islands Fisheries Science Center, Honolulu, Hawaii, United States of America; 4 Department of Biology, San Diego State University, San Diego, California, United States of America; 5 Woodrow Wilson School, Princeton University, Princeton, New Jersey, United States of America; 6 National Oceanic and Atmospheric Administration (NOAA), National Ocean Service, National Centers for Coastal Ocean Science-Biogeography Team and The Oceanic Institute, Waimanalo, Hawaii, United States of America; 7 Florida Museum of Natural History, University of Florida, Gainesville, Florida, United States of America; 8 Pacific/Remote Islands National Wildlife Refuge Complex, U.S. Fish and Wildlife Service, Honolulu, Hawaii, United States of America; 9 CORDIO East Africa, Mombasa, Kenya; 10 National Oceanic and Atmospheric Administration (NOAA), Joint Institute for Marine and Atmospheric Research and Pacific Islands Fisheries Science Center, Coral Reef Ecosystem Division, Honolulu, Hawaii, United States of America; 11 Smithsonian Tropical Research Institute, Balboa, Republic of Panama; 12 Centre d'Estudis Avançats de Blanes, Consejo Superior de Investigaciones Científicas (CSIC), Blanes, Spain; Centre for DNA Fingerprinting and Diagnostics, India

## Abstract

Effective conservation requires rigorous baselines of pristine conditions to assess the impacts of human activities and to evaluate the efficacy of management. Most coral reefs are moderately to severely degraded by local human activities such as fishing and pollution as well as global change, hence it is difficult to separate local from global effects. To this end, we surveyed coral reefs on uninhabited atolls in the northern Line Islands to provide a baseline of reef community structure, and on increasingly populated atolls to document changes associated with human activities. We found that top predators and reef-building organisms dominated unpopulated Kingman and Palmyra, while small planktivorous fishes and fleshy algae dominated the populated atolls of Tabuaeran and Kiritimati. Sharks and other top predators overwhelmed the fish assemblages on Kingman and Palmyra so that the biomass pyramid was inverted (top-heavy). In contrast, the biomass pyramid at Tabuaeran and Kiritimati exhibited the typical bottom-heavy pattern. Reefs without people exhibited less coral disease and greater coral recruitment relative to more inhabited reefs. Thus, protection from overfishing and pollution appears to increase the resilience of reef ecosystems to the effects of global warming.

## Introduction

Early historical accounts of coral reefs describe an abundance of sharks and other large fishes and luxuriant coral growth that seem incredible in the context of today's coral reefs and modern reef science [1]–[6]. Quantitative surveys conducted as recently as the 1980s report surprisingly high densities of predatory fishes (e.g., sharks and groupers) and corals; [7]–[9]). In contrast, most recent accounts of reefs largely describe dramatic declines of entire guilds of large fishes and corals [10]–[15] . The decline of large predators is believed to affect strongly patterns of trophic flow in marine communities [16], [17], and declines in coral cover have been linked to decreases in abundance and diversity of reef fishes [18], [19]. Nevertheless, we still lack a comprehensive, quantitative description of the structure and functioning of pristine coral reef communities.

Ecological baselines of the structure and functioning of ecosystems in the absence of human impacts can provide fundamental insights for conservation and restoration [20]. For example, historical reconstructions of the frequency of fires in temperate forests have revealed the unanticipated ecological consequences of modern fire suppression [21]. Knowledge of baseline fire conditions and the positive ecosystem services afforded by occasional fires have played an important role in forest restoration and management [22]. Protected areas also can be used as ecological reference sites [20], [23], [24]. For example, studies of wolves in Yellowstone National Park (a large, protected area) have helped to quantify the ecological roles filled by these top predators. Wolves provide a consistent source of carrion (from incompletely consumed prey carcasses) for scavenger species, dampening the increasing fluctuations of food availability caused by climate change [25]. Thus, insights into the baseline structure and functioning of ecosystems are critical for development of effective conservation and restoration programs [26].

Recent surveys of the fishes of uninhabited, remote coral reefs in the Northwestern Hawaiian Islands and the northern Line Islands [23], [27] strongly support historical reports of great fish abundance and predator domination that characterized coral reefs before extensive fishing efforts (see historical references for the Line Islands in Table S2). Comparisons of these data with neighboring, inhabited sites suggest that these coral reef assemblages have been altered by human activities, mostly fishing [23], [27]. However, most reef studies focus on individual taxa and do not provide community-wide descriptions of baseline conditions. With very few exceptions, studies of reef communities across gradients of human disturbance have been conducted on reefs that were previously severely degraded and are only now recovering to varying degrees due to deliberate protection from fishing [12], [28]–[30].

**Figure 1:**
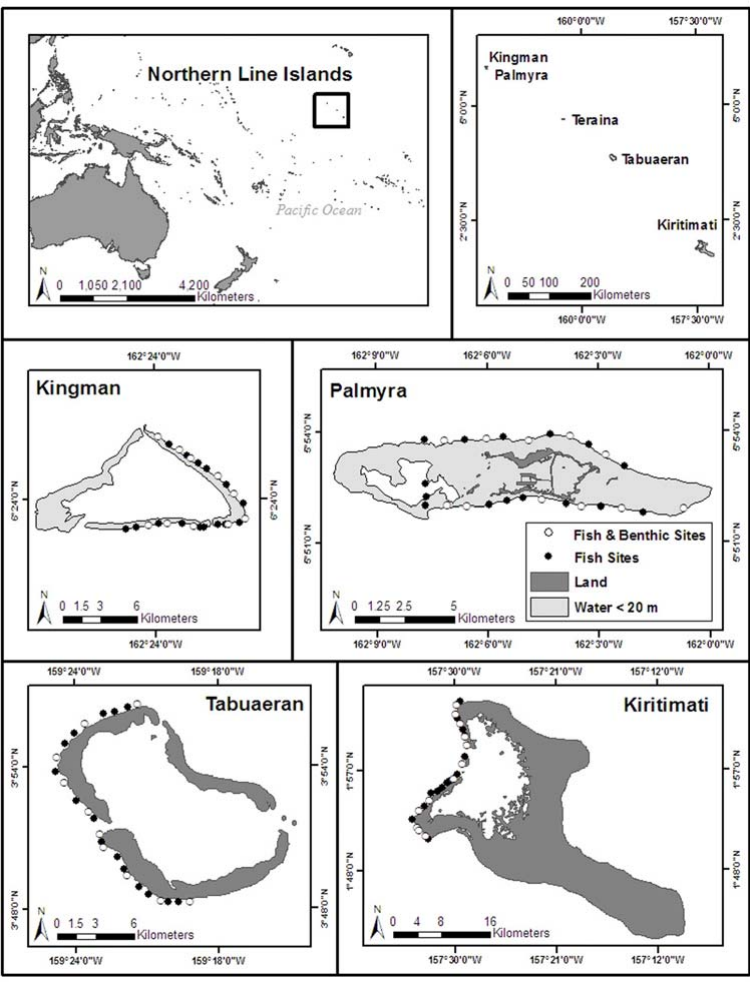
Location of study sites at Kingman, Palmyra, Tabuaeran and Kiritimati atolls in the Line Islands. Sites were located at semi-exposed fore reef habitats between 10 and 12 m in depth, and approximately one km apart from each other. Reef fishes were surveyed at all sites (n = 25 sites per atoll), and benthic communities at a subset of sites (n = 10–12 sites per atoll).

Palmyra and Kingman atolls in the northern Line Islands provide the opportunity to study reefs that have experienced minimal human impacts compared to most other reefs. Fishing pressure and oceanographic stress from upwelling or sea surface temperature anomalies are low in comparison with neighboring Tabuaeran and Kiritimati in the same biogeographic region (Fig. 1; also see Table 1 and Supplemental Data S1 for a detailed summary of anthropogenic and oceanographic conditions in the region). We surveyed in detail the fishes, corals, macroinvertebrates, and macroalgal assemblages across the four atolls to evaluate the effects of increasing human populations at Kiritimati and Tabuaeran relative to the baselines of Palmyra and Kingman. Detailed description of the microbial community is reported in a companion paper [31].

**Table 1:** Characteristics of the seven central Pacific atolls, including the four northern Line Islands and three additional equatorial atolls.

	Human involvement						Environment	
	Perimeter (km at 10 m isobath)	Population (2005 est.)	Pop. density (# [inhabited km reef]^−1^)	Fishing activities	Agricultural activities	Other activities	SST (°C)	Mean max. annual # of DHW
**Kingman**	34.4	0	0	Prohibited	-	-	27.9 (0.7)	1.32 (1.61)
**Palmyra**	40.8	20	0.5	Prohibited	Copra (historic)	Navy base (1940–46)	27.9 (0.8)	2.69 (3.76)
**Tabuaeran**	52.3	2,539	62.2	Subsistence	Seaweed, copra	Shipwrecks	27.5 (0.9)	3.32 (4.45)
**Kiritimati**	146.8	5,115	108.9	Subsistence/commercial	Seaweed, copra	Nuclear tests, shipwrecks	27.1 (1.1)	5.47 (6.48)
**Jarvis**	12.4	0	0	Prohibited	Guano removal	Small habitation (early 1900s)	27.0 (1.2)	6.99 (8.14)
**Howland**	8.9	0	0	Prohibited	Guano removal	Limited military (1930s–40s)	28.1 (1.0)	6.80 (6.49)
**Baker**	9.7	0	0	Prohibited	Guano removal	Limited military (1930s–40s)	28.1 (1.0)	6.82 (6.43)

	Environment (cont.)						Biota			
	Annual freq. of NOAA Level 2 bleaching events	Recent years of Level 2 events	Chl *a* (mg m^−3^)	*In situ* Chl *a* (mg m^−3^)	DIN (µM L^−1^)	SRP (µM L^−1^)	Biomass of total fishes (mT ha^−1^; w/percent piscivorous in brackets)	Number of fish species	Coral cover (%)	Number of coral species
**Kingman**	0%	-	0.10 (0.05)	0.046±0.005	1.00±0.14	0.107±0.007	5.3±0.7 [85%] b,c	232 {223, 244}	43.8±5.4 b,c	106 {101, 114}
**Palmyra**	14%	1994, 2002	0.10 (0.05)	0.069±0.013	1.75±0.44	0.120±0.012	2.6±0.3 [65%] b,c	234 {227, 244}	20.4±2.7 b,c	102 {93, 113}
**Tabuaeran**	19%	1994, 1997, 2002	0.15 (0.03)	0.146±0.030	2.51±0.25	0.197±0.016	1.7±0.2 [24%] b	250 {243, 260}	19.5±4.0 b	103 {97, 111}
**Kiritimati**	19%	1991, 1997, 2002	0.16 (0.02)	0.247±0.042	3.61±0.19	0.295±0.036	1.3±0.1 [15%] b	281 {273, 292}	15.0±2.3 b	83 {78, 90}
**Jarvis**	33%	1991, 1992, 1994, 1997, 2002	0.18 (0.02)	N/A	N/A	N/A	8.0±2.1 [74%] a	-	43.9±8.0 a	-
**Howland**	38%	1992, 1993, 1994, 2002, 2004	0.16 (0.05)	N/A	N/A	N/A	4.2±0.2 [54%] a	-	49.5±5.9 a	-
**Baker**	38%	1992, 1993, 1994, 2002, 2004	0.16 (0.05)	N/A	N/A	N/A	3.9±0.2 [46%] a	-	55.6±5.1 a	-

Quantitative environment and biota data are presented as means with variability presented as either standard error (reported with ‘±’), standard deviation (reported in parentheses), or 95% confidence intervals (reported in braces). Abbreviations are as follows: SST, Sea surface temperature; DHW, Degree heating weeks; Chl *a*, Chlorophyll *a*; DIN, Dissolved inorganic nitrogen; SRP, Soluble reactive phosphorous. Details on the estimations and calculations are presented in the Methods and Supplemental Data.

a Unpublished data from NOAA Fisheries, Pacific Islands Fisheries Science Center, Coral Reef Ecosystem Division and US Fish and Wildlife Service. These data were collected using similar methods as those used in this study.

b This study

c NOAA data showed a total fish biomass of 1020 g m^−2^ and 49% coral cover at Kingman, and 520 g m^−2^ and 45% at Palmyra. Differences in estimates of biomass across the different data sets may be due to a difference in sampling effort, higher variability in habitat types in the NOAA data, and changes over time.

## Results

### Reef Fishes

**Figure 2:**
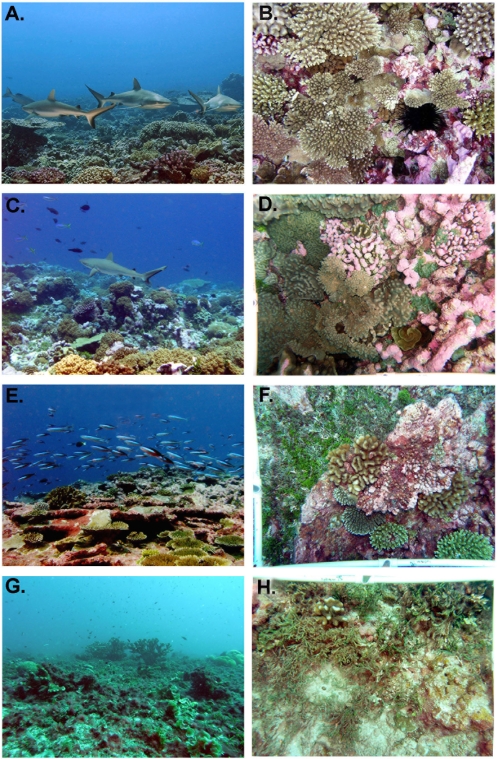
General aspect of fore reef habitats (left column) and representative 0.5-m2 photos of the bottom (right column) at Kingman (A–B), Palmyra (C–D), Tabuaeran (E–F), and Kiritimati (G–H), showing the degradation from a reef dominated by top predators and corals (Kingman) to a reef dominated by small planktivorous fishes and algae. Photo credits: A by Zafer Kizilkaya, B–H by Jennifer Smith.

**Figure 3:**
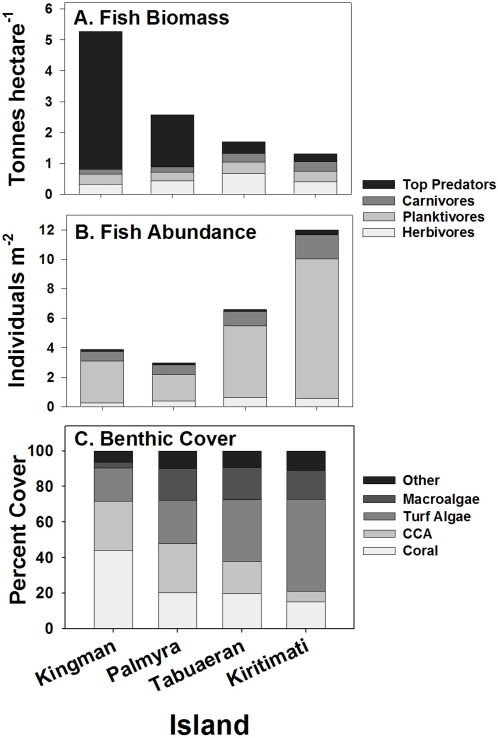
Fish biomass (A) and abundance (B), and cover of major benthic functional groups (C) across the northern Line Islands. Note that human impacts increase from left to right in this and subsequent figures. CCA = crustose coralline algae.

Biomass and abundance of reef fish varied greatly from Kingman to Kiritimati (Fig. 2). Total fish biomass decreased from 527 to 132 g m^−2^ (1-way ANOVA on atoll effect, F_3,97_ = 27.6, p<0.0001; Fig. 3A) whereas total abundance increased from 4 to 12 fishes m^−2^ (F_3,97_ = 69.2, p<0.0001; Fig. 3B). This contrasting pattern of fish biomass and abundance reflects a shift from dominance by a few large top predators at Kingman to many much smaller, lower trophic level consumers, especially planktivores, at Kiritimati. Species richness increased from Kingman to Kiritimati, generally tracking the increase in fish abundance (Table 1, Fig. 3B).

Top predators accounted for 85% of total fish biomass at Kingman, and decreased to 19% at Kiritimati (Fig. 3A). The dominant predators at Kingman were snappers, jacks and sharks (median total length = 33 cm, maximum = 200 cm) versus small groupers (principally Epinephelinae; median = 13 cm, maximum = 65 cm) at Kiritimati. Sharks comprised 74% of the top predator biomass (329 g m^−2^) at Kingman and 57% at Palmyra (97 g m^−2^), whereas they were virtually absent at Tabuaeran and Kiritimati. Thus, the typical fish biomass pyramid observed at most reefs around the world today [12], [32], [33], including those of Tabuaeran and Kiritimati, is inverted at Kingman and Palmyra. Inverted biomass pyramids of fishes have only been documented elsewhere in the Northwestern Hawaiian Islands [23]. Thermodynamic constraints, however, require that inverted biomass pyramids be supported by bottom-heavy pyramids of production [34], [35]. This suggests that turnover rates of predators are much lower than of their prey, and that trophic efficiency is high at all levels. Such dramatic alterations of rates and pathways of trophic flow have been afforded little research effort to date (see notable exception in [36]).

The structure of lower trophic level fish assemblages also changed across the gradient. Carnivores (principally predators upon invertebrates) had lower biomass at Kingman and Palmyra than at Tabuaeran and Kiritimati (F_3,97_ = 68.1, p<0.0001). Small planktivores only a few centimeters in length were the most numerous fish at all atolls, especially at Kiritimati, but planktivores accounted for only 6% of total fish biomass at Kingman compared to 26% at Kiritimati (Fig. 3). The biomass of herbivorous fishes was greatest at Tabuaeran (F_3,97_ = 8.4, p<0.0001) but did not differ among the other atolls. The mean per capita mass of herbivores was smallest at Kiritimati (F_3,97_ = 8.9, p<0.0001), driven largely by a 60-fold increase in biomass of small-bodied, territorial damselfish from Kingman to Kiritimati.

The changes in reef fish assemblage structure are best described as a response to increased fishing pressure from Kingman to Kiritimati (Supplemental Data). Fishing pressure tends to disproportionately reduce densities of longer-lived, larger-bodied individuals [37], [38], which are frequently from higher trophic levels [39]. Recent studies indicate that the loss of live coral cover, for example due to bleaching, can reduce the density of small-bodied fishes that seek food and/or shelter in, or recruit to, the living coral matrix [11], [18]. In the northern Line Islands, in contrast, decreasing coral cover was associated with an increase in the abundance of smaller fishes (Fig. 3), suggesting that the effects of coral loss on fish assemblage structure are secondary to (although likely reinforce) fishing impacts.

### Benthic Community

Benthic community structure shifted from domination by reef-building stony corals and crustose coralline algae (CCA) at Kingman and Palmyra to domination by macroalgae (including species of *Halimeda, Caulerpa, Avrainvillea*, *Dictyosphaeria*, and *Lobophora*) and algal turfs at Tabuaeran and especially Kiritimati (PERMANOVA, F = 14.1, p = 0.001) (Figs. 2 and 3C). Stony corals plus CCA strongly dominated the reefs at Kingman (71% cover; Fig. 3C) with numerous large coral colonies (primarily plate-forming and branching *Acropora* spp.). Cover of stony corals plus CCA dropped to 48% at Palmyra, 38% at Tabuaeran, and 21% at Kiritimati (Fig. 3C). Benthic community composition was much more variable at Tabuaeran than the other atolls, ranging from sites dominated by CCA and *Acropora* corals (maximum 63% combined cover) to sites dominated by fleshy algae (79% combined cover of turf and macro-algae). However, turf algae were the most common benthic group overall (36% cover; Fig. 3C), whereas dead corals carpeted by turf and macroalgae more uniformly dominated the reefs at Kiritimati (68% mean combined cover).

**Figure 4:**
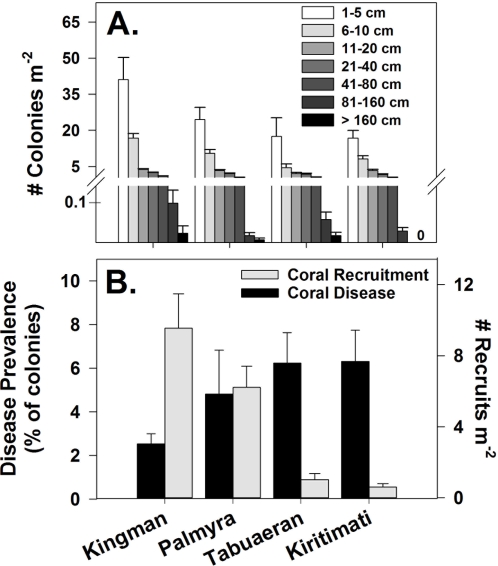
Size frequency distribution of corals (A), and coral disease prevalence and recruit density (B) across the northern Line Islands. Because recession in adult corals can often lead to fragmentation and the development of many small colonies that may have all been part of one larger colony, recruits are defined here as colonies less than 5 cm in diameter from coral taxa demonstrating essentially unidirectional growth (i.e., Acropora, Pocillopora and Fungiids), and thus represent individuals known to be young relative to larger individuals. Note that there were no colonies larger than 160 cm at Kiritimati.

Coral density (numbers of colonies m^−2^) tracked coral cover and was highest at Kingman and lowest at Kiritimati (ANOVA, F_3,304_ = 3.1, p<0.03) (Fig. 4A). However, the size frequency distributions of corals were statistically indistinguishable among atolls (size class×atoll interaction, F_3,300_ = 1.8, p>0.1). Nevertheless, we found no colonies greater than 160 cm at Kiritimati, although there were numerous smaller colonies apparently resulting from the partial mortality of previously larger diseased or degenerating colonies. Coral species richness across the sampled area decreased from Kingman to Kiritimati, paralleling the decrease in density (Table 1).

We surveyed 24 species of mobile macroinvertebrates and 21 morphospecies of sessile (non reef-building) macroinvertebrates. In general, exposed and conspicuous macroinvertebrates were uncommon at all atolls with no obvious correlation with human abundance (Table S3). Sea urchins were more abundant at Kingman (24.6 individuals 100 m^−2^) than at any of the other atolls (range = 0–8.2 100 m^−2^; non-parametric median test, p<0.0001) primarily due to *Echinothrix diadema* (p<0.0001) (Tables S3 and S4). Holothurians (1.1 individuals 100 m^−2^; p = 0.03) and giant clams (*Tridacna maxima*; 0.75 individuals 100 m^−2^; p = 0.018) were also more abundant at Kingman than elsewhere. These animals are targeted for consumptive harvest at Kiritimati (Tables S3 and S4).

### Coral Recruitment and Disease

Numbers of small colonies (1–5 cm) of *Acropora*, *Pocillopora* and fungiids, taxa that could be definitively identified as recruits, were more than 6 times higher at Kingman and Palmyra than at Tabuaeran and Kiritimati (Fig. 4; ANOVA, *F_3,40_* = 25.9, p<0.001; Fig. 4A). These differences were robust after correcting for inter-atoll differences in total coral density (i.e., densities of recruits standardized by the mean atoll-specific total densities of colonies<5 cm; *F_3,40_* = 15.8, p<0.001). Prevalence of coral disease showed the opposite pattern, and was lowest at Kingman and highest at Kiritimati and Tabuaeran (Kruskal Wallis test; H = 8.0, df = 3, p = 0.04) (Fig. 4B). Notably, the pattern of disease prevalence paralleled that of microbial densities both on the benthos and in the water column (see [31] for a more detailed treatment of these findings).

### Coral Reef Community Structure: a Food-Web Perspective

**Figure 5:**
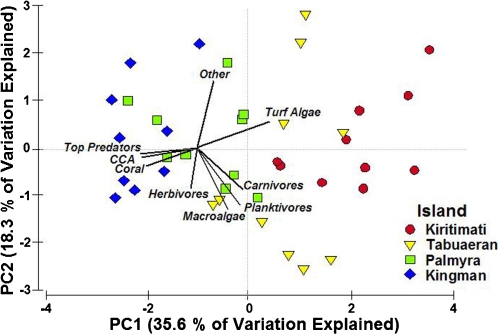
Principal component analysis (PCA) of major fish and benthic groups from all sites with photoquadrat data (n = 10 per atoll, except Kingman with n = 9). Atolls cluster in sequence along the first principal component axis (PC1), shifting from left to right from Kingman to Kiritimati. The major loadings on PC1 include biomass of top predatory fish, coral and CCA cover (to left, i.e., decreasing disturbance) and turf algal cover (to right, i.e., increasing disturbance). Note that herbivore biomass, the putative intermediate in models of simple trophic cascades, is orthogonal to the dominant axis correlated with apex predators.

We used principal component analysis (PCA) to explore the correlations among density estimates for dominant functional groups (four fish guilds and five benthic types) for all sites at which both benthic photoquadrat and fish census data were collected (Fig. 5). The first two principal component axes (PC1 and PC2) described over 50% of the variation in the data and thus provide insights into the dominant correlations among these data [40]. PC1 paralleled the gradient of atolls, with sites from each atoll clustering together and organized in sequence (left to right from Kingman to Kiritimati, Fig. 5). The major loadings of PC1 were biomass of top-predatory fish, coral cover, and CCA cover in the direction of Kingman versus turf algal cover in the opposite direction. Loadings for biomass of non-predatory fishes were generally orthogonal to the loading for predator biomass. As such, there was no evidence of a simple trophic cascade within the reef food web, contrary to some theoretical predictions [41], [42].

The mechanisms leading to the marked association between high coral cover, low cover of turf, and abundant top predators across sites (as noted along PC1) are unclear. In contrast to other observational studies [43], there was no obvious correlation between the abundance or biomass of herbivorous fishes and benthic community structure. Herbivore populations remained relatively constant (but generally high in comparison to other reefs [23]) across the atolls. Systematic shifts in the size-structure of herbivorous fish (smallest on Kiritimati) or in the relative abundance of functional types (increase of territorial damselfish from Kingman to Kiritimati) may have altered patterns of herbivory contributing to the observed differences in benthic structure. Further, grazing by invertebrates may also help to explain the differences in algal communities across atolls as sea urchins were most abundant on Kingman. An alternative explanation for the low macroalgal cover and high coral cover (and recruitment) at Kingman is that higher coral cover concentrates the grazing of herbivorous fish. Most herbivorous fish in the Pacific feed primarily on algae instead of corals [33], causing the intensity of herbivory on non-coral substrata to be greater at sites where coral cover is high [44]. However, if it is assumed that each atoll had comparable coral cover prior to human arrival and recent climate change, this mechanism of concentrating herbivory is inadequate to explain the original loss of live coral cover at Tabuaeran and Kiritimati.

### Diversity and Disturbance

Changes in species diversity were inconsistent across taxonomic groups: the number of coral species counted decreased from Kingman to Kiritimati whereas the number of fish species increased (Table 1). Decreasing diversity with increasing human disturbance is commonly assumed, and has been reported at other sites in the past for both corals [45] and fishes [46], [47]. In a recent study, Tittensor et al [47] report decreasing fish diversity from Palmyra to Kiritimati (they did not sample Kingman), which is in contrast to our findings. However, their analysis is not directly comparable because it was done at the family level and different habitats were analyzed on different atolls (back-reefs on Palmyra, fore-reefs on Kiritimati and Tabuaeran); they also surprisingly failed to observe significant differences in benthic composition among atolls, in contrast to the strong differences documented here (Fig. 3C).

Both intermediate disturbance models [48], [49] and abundance-based models [50], [51] are consistent with the diversity patterns we observed. A recent meta-analysis documented an increase in fish species richness with moderate declines in coral cover that shifted to a decrease in richness with more severe declines of coral [52], consistent with the model of intermediate disturbance. But regardless of the underlying cause, our results suggest that diversity alone is not a clear indicator of ecosystem health or condition, particularly across gradients that include undisturbed habitats like Kingman.

### Local Human Impacts and Oceanography across the Northern Line Islands

Kingman, Palmyra, Tabuaeran, and Kiritimati vary in human population size from uninhabited to moderately populated (Table 1). Unpopulated Kingman lacks permanent emergent land, is protected as a National Wildlife Refuge by the United States Fish and Wildlife Service (USFWS), and is not fished. Palmyra Atoll was physically altered by extensive dredging during the 1940s and was historically fished, but currently is fully protected from fishing by the USFWS. The camp at Palmyra has a maximum population of 20 people with modern sewage treatment. Tabuaeran has a permanent, growing population, estimated at 2,500 people in 2005, that subsists on fishing and has no sewage treatment. Kiritimati has an even more rapidly growing population, estimated to be 5,100 in 2005, that subsists primarily on fishing and lacks a functioning waste disposal system. Kiritimati also supports a commercial fishing operation for both food and aquarium fish (see the Supplemental Data S1 for further detail). Based on fisheries surveys, however, Tabuaeran and Kiritimati experience appreciable fishing with subsistence harvest yielding 20 t yr^−1^ and 408 t yr^−1^, respectively, directly from the reef. Over 50% of the reef fishery is composed of predatory species. Comparing these estimates with our *in situ* counts (Figure 3), this yield is analogous to the complete removal annually of predatory fish from 27 and 800 ha of fore reef at Tabuaeran and Kiritimati, respectively.

We did not measure nutrient flow from human settlements to the reef at Tabuaeran and Kiritimati, but a simple calculation suggests that anthropogenic runoff is of minor importance. Industrial agriculture and manufacturing are absent and the primary input is sewage (Supplemental Data S1). Assuming that the average person excretes 10 g of nitrogen per day [53], the per capita annual nitrogen input to the watershed would be approximately 3.5 kg yr^−1^. This is <5% of the typical per capita impact of industrialized, non-insular populations [54], which suggests a weak effect of direct input by humans to the observed gradient in nutrient concentrations across the archipelago (Table 1).

The gradient in human habitation and fishing in the northern Line Islands parallels that of several oceanographic factors, confounding interpretation of the observed patterns in the biota and human populations. Sea surface temperature (SST) generally increases with northern latitude in the region, although annual mean SST varies by just 0.8°C between the atolls (Table 1, Figure S1). The more equatorial atolls, however, experience greater year-to-year variability in SST, mainly due to El Niño Southern Oscillation (ENSO) events. Mean annual accumulation of degree heating weeks (DHW, see Methods for definition) increases approximately 4-fold from Kingman to Kiritimati (Table 1). Thus, reefs of Kingman have experienced significantly fewer large-scale bleaching episodes than those of Kiritimati (sensu [55]–[57]). There is also an increase in productivity from north to south associated with stronger upwelling [58], [59]. Mean concentrations of total dissolved inorganic nitrogen (DIN), soluble reactive phosphorous (SRP), and chlorophyll *a* each increase appreciably from Kingman to Kiritimati (Table 1). Increase in nutrient delivery rates would favor macroalgal growth relative to coral growth [60], or enhance fish (predominately planktivore) population sizes [61].

Historical reports and biogeographical comparisons, however, suggest that differences in oceanography alone are insufficient to cause the observed differences in fish and benthic communities among the atolls. The earliest historical descriptions of Kiritimati and Tabuaeran document an enormous abundance of sharks and other large fishes (Table S2) that persisted until the early to mid 20^th^ century when declines became apparent. As recently as 1997, fish biomass at Kiritimati was double that observed in our study and was comprised of over 30% top predators [62], suggesting that large declines in the fish assemblage have occurred within just ten years as the human population rapidly increased due to deliberate relocation (Supplemental Data S1; Figure S1). Thus, the low fish biomass at these atolls most likely is due to fishing here, as in many places elsewhere [39], [63], [64].

Coral cover also decreased greatly at Kiritimati, Tabuaeran, and Palmyra between 1997 and our surveys in 2005 [61], [65], but remained relatively constant at Kingman [61]. These declines likely reflect effects of high bleaching stress associated with sustained periods of anomalously high seawater temperatures in the late 1990s and early 2000s (Table 1) [61]. However, comparison with atolls near to the northern Line Islands suggest that human activities have greately exacerbated the effects of high temperature at Tabuaeran and Kiritimati. Uninhabited and protected Jarvis, Howland, and Baker atolls lie 1° to 1.6° to the south of Kiritimati and exhibit relatively higher chlorophyll *a* concentrations and more frequent SST anomalies associated with coral bleaching (i.e., NOAA Level 2 events; Table 1) [61], [66]. Extensive episodes of upwelling also have been documented in detail at Jarvis [67]. Nevertheless, these equatorial atolls support very high biomass of fishes dominated by apex predators and high live coral cover comparable to those at Kingman (Table 1) [61]. Because of the remoteness of the atolls, monitoring of the biota has been infrequent and the history of coral bleaching has not been studied in detail. But the fact that the coral cover at these atolls has remained high despite such thermal stress suggests that the corals have survived or have recovered from warm-water anomalies (consistent with models of ecological resistance or resilience, respectively) at uninhabited Jarvis, Howland and Baker, but not at Kiritimati and Tabuaeran where fishing effort and human population density are the highest in the region.

Combining data from our study with those from Jarvis, Howland and Baker (Table 1), we found no significant correlation between fish biomass or coral cover versus any of the oceanographic parameters (Frequency of Thermal Stress, SST, DHW, Chl *a*, DIN, SRP). Most strikingly, there were no correlations between fish or corals and either the maximum number of DHW per year (Pearson Correlation for fish: *r* = 0.44, *t* = 1.08, p = 0.84; and for coral: *r* = 0.28, *t* = 0.65, p = 0.73) or the frequency of thermal stress (NOAA level 2) that is commonly associated with serious coral bleaching events (fish: *r* = 0.23, *t* = 0.52, p = 0.69; coral: *r* = 0.49, *t* = 1.26, p = 0.87).

It is interesting that the concentration of nutrients (DIN and SRP) around all four of the northern Line Islands surveyed are higher than the hypothesized thresholds for outbreaks of macroalgal blooms [68] and are well within the range for polluted Florida Bay (e.g., 0.7 to 10.7 µMol for DIN) [69]. Nevertheless, macroalgal blooms are absent at Kingman and Palmyra, which suggests that grazing activity on unpopulated reefs may control macroalgal abundance even at high inorganic nutrient concentrations.

We propose a model of reef degradation consistent with our data and analogous to Birkeland's concept of ratcheting down coral reefs, where multiple, interacting effects of anthropogenic and natural stressors lead to the demise of reef health [70], [71]. In the northern Line Islands the combined effects of local human impacts (predominantly fishing), global human impacts (global warming), and natural oceanographic variation have contributed to the observed patterns. The death of many corals from Kiritimati and Tabuaeran between 1997 and 2005 was most certainly caused by warm water events in the late 1990s and early 2000s. However, the magnitude of and the lack of recovery from these mortality events was likely influenced by anthropogenic changes in the local reef community. Based on the historical and geographic comparisons outlined above, we suggest that reef degradation in the northern Line Islands started because of and was enhanced by local anthropogenic stress.

### Conclusion

Fish biomass and the proportion of apex predators at Kingman atoll are greater than previously described from any coral reef ecosystem [15], [27] and was associated with high cover of reef-building corals and crustose coralline algae, abundant coral recruits, and low levels of coral disease. More detailed, long-term observations and experiments are needed to more firmly establish cause and effect. But it is already apparent that reef communities with relatively intact food webs like those at Kingman, Jarvis, Howland, and Baker are the best available baselines for Pacific reefs, with biomass of top predators higher than in other well-protected areas such as the Great Barrier Reef in Australia [72], Kenya [15], or the Northwestern Hawaiian Islands [23]. Moreover, these uninhabited reefs appear to retain greater capacity to survive or recover from major episodes of coral disease or bleaching, whereas reefs with highly altered food webs like Tabuaeran and Kiritimati do not.

Thus, local protection from overfishing and pollution may enhance ecosystem resilience to warm episodes and coral bleaching that result from global warming. To test this we need to determine how do coral recruitment, growth, and survivorship respond to changes in local community structure due to fishing, and how do these responses interact with episodes of warming measured by DHW. We also need to determine how fish productivity, i.e., the key currency of fisheries management, varies with changes in food web structure such as those observed between Kingman and Kiritimati. The only way to answer these questions is by investigation of reefs like the northern Line Islands that have remained remarkably intact in comparison to the global norm. They are among the only baselines that remain.

## Materials and Methods

Surveys of macroflora and macrofauna were conducted using SCUBA-assisted coral reef assessment techniques in August-September, 2005. Approximately 20 km of coastline were surveyed per atoll in the semi-exposed leeward fore reef habitats between 10–12 m depths. We chose to compare the leeward side of the atolls for logistical reasons and because human activities are concentrated on the leeward side near human settlements and anchorages. However, windward and leeward are not as clearly defined around the smaller Palmyra and Kingman atolls so we sampled around the entirety of these atolls to maintain consistent areal coverage within the specified depth range.

### Reef Fishes

Surveys were conducted by two teams of paired divers, with four divers (ED, AF, ES, SS) rotating between teams to distribute individual biases. Stations were spaced about a kilometer apart at random locations. At each station, one team of tandem-paired divers tallied all fishes as they were encountered within fixed-length (25-m) strip transects whose widths differed depending on direction of swim. Transect bearings were determined haphazardly along isobaths (between 10 and 12 m depth). Each diver was responsible for one-half of the areas surveyed, as follows: large-bodied vagile fishes ≥20 cm total length (TL) were tallied within an 8-m wide strip (two 4-m wide swaths separated by 1 m) surveyed on an initial “swim-out” as the transect line was laid. Small-bodied, less vagile and more site-attached fish <20 cm TL were tallied within a 4-m wide strip surveyed on the return swim back along the laid transect line. Fishes were recorded by species or lowest recognizable taxon. Tallies were binned by 5-cm TL class. Three transects, each separated by about 10-m distance from its neighbor, were surveyed at each station. Thus, at each station, the densities of large-bodied fishes were estimated within a 600 m^2^ (3×25×8 m) area, and the densities of small fishes within a 300 m^2^ (3×25×4 m) area. Additional species richness data were recorded to complement those recorded on transects. Species presence was tallied within 3,000 m^2^ (100-m long by 30-m wide) areas searched by 1-way zigzag swims centered on the transect lines.

Transects provided the input to estimates of species- and size-specific numerical densities. Various published [73], unpublished (JD Parrish, US Geological Survey, Hawaii Cooperative Fishery Research Unit), and web-based [33] sources provided the length-weight regression parameters necessary for converting numbers to biomass. Density and biomass were standardized to one square meter. No differences in total fish biomass were apparent among the various teams of paired divers (2-way ANOVA—team effect: F_3, 93_ = 1.66, p = 0.18; team×atoll interaction effect: F_9, 82_ = 2.09, p = 0.04 ns). Second, there were no differences among teams in recorded composition of fishes; species density (richness per station) averaged 100±5 species per station for all teams (2-way ANOVA—team effect: F_4, 93_ = 2.24, p = 0.07; team×atoll effect: F_10, 83_ = 1.65, p = 0.11).

Numerical density and biomass of major fish trophic groups were compared among reefs by k-sample median tests [74] and ANOVA. Analysis routines were applied using SAS v. 9.

### Corals

Corals were surveyed in four ways to characterize their abundance, diversity, size distribution, and health. (i) Coral density and size was measured in two 25×2 m transects (100 m^2^ total area) per site; the identity (at least to genus level) and size of all corals with colony center within 1m of the transect were measured. (ii) Percent cover was estimated using photoquadrats (see *Benthic cover* below). (iii) We quantified coral health by measuring pathologies and magnitude of disease, bleaching, and/or drastic predatory damage recorded (see *Coral disease* below). (iv) In the 60×20 m area including the transects, divers (JM and DO) swam to develop a site-specific diversity list to the species level.

### Benthic Cover

Quantitative assessments of the benthos were made using the photoquadrat method [75]. At each site two 25 m transects were placed on the benthos parallel to shore, 25 m apart and at a constant depth of 10–12 m. Ten points were randomly selected and surveyed per transect. At each point a photograph was taken using an Olympus 7070 digital camera that was connected to a quadpod (1 m high) and a frame (0.9×0.6 m or 0.54 m^2^). During surveys notes were made for each quadrat and collections were made for organisms that were unidentifiable in the field. Upon return to shore all photographs were edited using Adobe Photoshop v 7.0. Image analysis was completed using the program Photogrid 1.0. For each photograph 100 points were randomly generated and the organism under each point was identified. A total of 10 benthic sites were surveyed for each atoll. All organisms were identified to the finest level of resolution possible (genus level for hard and soft corals, functional group for algal turfs and crustose coralline algae, and species level for macroalgae and macroinvertebrates when possible).

The structure of the benthic community was compared among reefs by PERMANOVA [76], and the cover of individual functional groups of organisms by ANOVA. To test for differences in the density of coral recruits (small colonies [1–5 cm] of *Acropora*, *Pocillopora* and fungiids, taxa that could be definitively identified as recruits) we performed ANOVA on log-transformed data. To compare the standardized coral recruit density among atolls (densities of recruits standardized by the mean atoll-specific total densities of colonies <5 cm) we performed ANOVA on arcsine square root transformed data.

### Coral Disease

Surveys describing the health status of corals were conducted on two, 2×20 m belt transects at 10 sites on each of the four atolls. Coral colonies were examined for gross morphological signs of stressors and placed into either a known disease, compromised or predation categories. The disease categories included, White syndrome, Skeletal Eroding Band, Brown Band, Black Band and other cyanobacteria (descriptions found in [77]). Compromised categories included signs of coral tissue necrosis that are not a formally recognized disease state (such as a degenerative syndrome, where partial mortality was caused by a combination of sedimentation, increased mucus production, and the presence of low numbers of cyanobacteria); algal interactions with corals that cause tissue bleaching and erosion; bleached white patches, which were bleached but had intact tissue on coral colonies and pink coloration (which suggests a stress response by the coral, mostly present in the family Poritiidae). Predator feeding scars from *Drupella* species and *Acanthanster planci* were recorded, but were not analyzed in this study. Coral showing signs of either disease or compromised health were used to analyze the relationship between the number of unhealthy and healthy corals on coral reefs associated with different levels of human activity. Because of logistical constraints, surveys describing the health status of the corals and those enumerating total coral numbers were conducted by separate researchers on the same transects. The transects focusing on disease signs were 5 m shorter than transects conducted to enumerate the total number of corals at each site (i.e. the first 20m of the 25m benthic transect). Therefore, the prevalence of unhealthy corals was calculated on a m^2^ basis by dividing the number of unhealthy colonies by the total number of coral colonies. To test for differences in the incidence of coral disease among atolls we used a non-parametric Kruskal-Wallis test.

### Mobile and sessile (non-coral) macroinvertebrates

Population density and species richness of mobile macroinvertebrates were determined within one 60×2 m belt transect at each of the 10 benthic stations on each atoll (except at the first station in Kiritimati, where a 1m wide transect was used). Mobile macroinvertebrates were defined as species that live unattached and grow to >5 cm, and comprised 24 species of echinoderms (asteroids, echinoids, holothurioids), mollusks (gastropods, bivalves, and cephalopods), and crustaceans (lobsters). Only visible (exposed or partially exposed) animals were counted. Species richness of sessile macroinvertebrates other than scleractinian corals was determined around the same belt transect used for mobile macroinvertebrates. Both species within the belt transect, as well as those encountered around the belt transect within a 30-minute total search time were recorded. Sessile macroinvertebrates were defined as species that live attached and grow to >5 cm, and comprised 21 species and forms of anthozoans (octocorals, zoanthids, actiniarians, and antipatharians), and sponges. *Sinularia* soft corals were differentiated into morphospecies based on growth form; all other anthozoans and sponges were differentiated to species. Only visible (exposed or partially exposed) animals were recorded. Numerical density of major species and taxonomic groups were compared among reefs by k-sample median tests [74].

### Structure of coral reef communities

To explore the gradients in coral reef community structure (abundance of functional groups) among sites and atolls we performed a principal components analysis. Data were normalized, following arcsine square root transformation for benthic percent cover and natural logarithm transformation for fish biomass, and standardized.

### Species richness of fishes and corals

Total species richness estimates were compared among atolls. For fishes and for corals, richness was estimated using presence/absence data from each station. Incidence-based coverage estimators were used to account for differing abundances across atolls, providing an extrapolated estimate of the total number of species present in the sampled area [78].

### Oceanographic data

Mean and standard deviation of sea surface temperature (SST) were calculated from monthly mean SST estimates for the 50km water pixel including each atoll using US National Oceanic and Atmospheric Administration (NOAA) Coral Reef Watch data from 1985–2005.

The annual accumulation of degree heating weeks (DHW) is determined from these twice-weekly SST data. One DHW is equal to one week of SSTs that are one degree greater than the mean temperature of the warmest month in the climatology (i.e., the historical mean for July in most of the Line and Phoenix Islands). The total DHW accumulation for a given year is the accumulation of positive SST anomalies over a rolling 12 week time period; only anomalies in excess of 1°C were included, because smaller SST spikes are believed to be insufficient to cause stress on corals (i.e., consecutive weekly anomalies of 1.0, 1.5 and 0.8 results in a total DHW value of 2.5, because the third value is less than one). Notably, El Niño Southern Oscillation (ENSO) anomalies are the common cause of maximum DHW episodes. Because ENSO events typically occur during the boreal winter (December–January) in the region, we calculated the annual maximum number of DHW for 12 mo periods from July in one year until June in the following year.

Bleaching Alert Level 2 is the highest level of coral bleaching warning issued by NOAA and is defined as a period with ≥8 maximum DHW. We computed the frequency of Level 2 bleaching events as the percentage of years from 1985–2005 with maximum DHW at or above this threshold. We present individually the years since 1990 that have reached this threshold.

The chlorophyll *a* data used in this study were acquired using the GES-DISC Interactive Online Visualization ANd aNalysis Infrastructure (Giovanni) as part of the NASA's Goddard Earth Sciences (GES) Data and Information Services Center (DISC). The OBPG (Ocean Biology Processing Group) MODIS/Aqua monthly global 9-km products were subdivided into 2°×2° boxes surrounding each atoll creating area-averaged values for each box. Monthly chlorophyll a and standard deviation products were averaged over the available time period (July 2002 through October 2006) to calculate mean chlorophyll a concentration (mg/m^3^) and standard deviations for each of the atolls. Kingman and Palmyra were calculated using the same 2°×2° bin because of their proximity to each other; Howland and Baker were calculated the same way.

### Water chemistry

At each atoll water samples were collected from 4 (5 at Palmyra) evenly distributed stations at approximately the same time in the morning on each occasion. At each station a total of 8 samples were taken using diver-adapted polycarbonate 2-liter Niskin bottles. Water was transferred back to the OR/V White Holly and processed within 1 hour of collection. The Niskin bottles were connected directly to a positive-pressure filtration system minimizing external contamination. All fixtures were made from polycarbonate or silicone. Acid washed tubing and filter cassettes were flushed with 200 ml of the sampled water before sample collection began. Samples were collected under positive pressure by applying 3 PSI of pressure to the Niskin bottles using a SCUBA tank.

Water was collected for inorganic nutrient analysis by filtering through 0.2 µm Nuclepore Track-Etch membrane filters (Whatman). Fifteen ml of filtrate was collected in HDPE scintillation vials with cone-shaped plastic lined lids (Fisher Scientific) after rinsing both the bottles and lids 3 times with filtrate, and then stored at −20°C. Analysis of inorganic nutrients (nitrate, nitrite; ammonium and ortho-phosphate) concentrations was carried out by the Marine Science Institute's Analytical Lab, University of California, Santa Barbara, California, USA using a QuikChem 8000 flow injection analyzer (Lachat Instruments, Wisconsin, USA). Data for nitrogen are presented as dissolved inorganic nitrogen (DIN), i.e., the total of ammonium and Nox (i.e., nitrate+nitrite). Data for phosphorus are presented as total soluble reactive phosphorus (SRP). Means and standard errors of nutrient concentrations were computed across stations for each atoll.

Water was also collected for chlorophyll a analysis. Triplicate 500 mL samples were filtered onto 25 mm GF/F (Whatman) and immediately stored at −80°C. Within two months, filters were extracted in absolute methanol for 1 h [79] and chlorophyll a concentrations measured on a Turner Designs (model 10–005R) fluorometer. Means for each station and standard errors among stations were determined for each atoll.

## Supporting Information

Supplemental Data S1 The gradient of human disturbance in the northern Line Islands: Population, fishing, and waste.
(0.05 MB DOC)

Table S1 Aquarium reef fish catch for export at Kiritimati (April–December, 2005).
(0.03 MB DOC)

Table S2 Historical accounts of shark abundance in the Line Islands.
(0.05 MB DOC)

Table S3 Non-coral invertebrate abundance on the northern Line Islands.
(0.12 MB DOC)

Table S4 Non-coral invertebrate data analysis: Median test for comparison of species abundance between atolls.
(0.05 MB DOC)

Figure S1 Population data for the northern Line Islands
(0.05 MB TIF)

Figure S2 Total annual reef fish catch at Kiritimati
(0.68 MB TIF)

Figure S3 Annual Fish Catch for Export on Kiritimati
(0.66 MB TIF)

Figure S4 Map of the central Pacific, including atolls of the Line and Phoenix Islands. Colors reflect mean sea surface temperatures for August 2005. Note the latitudinal gradient of temperature determined by meeting of the equatorial current and countercurrent
(3.43 MB TIF)
