# Supplementary material for: Baselines and Degradation of Coral Reefs in the Northern Line Islands
Source: PLoS One. 2008 Feb 27;3(2):e1548. doi: 10.1371/journal.pone.0001548 (PMC2244711; doi:10.1371/journal.pone.0001548)
Supplement: Supplemental Data S1 — The gradient of human disturbance in the northern Line Islands: Population, fishing, and waste. (0.05 MB DOC) [file pone.0001548.s001.doc]

**Supplemental Data S1**

**The gradient of human disturbance in the northern Line Islands: Population, fishing, and waste.**

Population

All of the Line Islands were uninhabited at the time of European discovery in the late 18^th^ century. However, there is evidence that Tabuaeran had some permanent populations that also made periodic trips to Kiritimati from AD 1200-1600 (*S1*, *S2*). After 1800, Tabuaeran was the first of the northern Line Islands to be occupied in 1820 (*S3*-*S4* cited in *S5*). However, the first occupation by Europeans and Hawaiians only lasted two years. Tabuaeran and Kiritimati experienced many successive short occupations during the first half of the 19^th^ Century (*S6*-*S8* cited in *S5*, *S9*-*S14*). By the 1850s, they were more or less occupied for the purposes of establishing copra plantations and mining phosphate (*S6-S8* cited in *S5*, *S13*). European entrepreneurs brought laborers from Manihiki (*S13*), the Society Islands (*S6* and *S17* cited in *S5*, *S15*), and the Gilbert Islands (*S13*). Some of these laborers were on short-term contracts and did not become permanent residents of the Line Islands. There was high turnover in the leases of the islands and brief periods with little to no occupation. The building of a relay station for the British Commonwealth’s transpacific communication cable brought a small permanent population to Tabuaeran from 1902-1963 (*S10*).

World War II had the most rapid and dramatic effect on populations at Palmyra and Kiritimati (*S5, S11*). American troops were garrisoned on both Palmyra and Kiritimati during the war. The military base on Palmyra was able to accommodate 6,000 people, although the military personnel left the island after WWII (*S5*). Aside from the activity associated with WWII, Palmyra only experienced periodic visits during the 1900s (*S16*). Kiritimati, however, was continuously occupied and was used for high-altitude nuclear testing by both the United States and Great Britain during the cold war (*S5*).

Most recently, Kiritimati and Tabuaeran have been the target of resettlement programs aimed at stimulating economic growth and relieving population pressure on South Tarawa, the capital of the Republic of Kiribati, which lies approximately 3,300 km west in the Gilbert Islands (*S17-S18, S19*). In 1988, a resettlement program brought people from Tarawa to Tabuaeran. The program was abandoned in 1992 due to considerations for the carrying capacities of the islands. Now, Kiritimati is the primary focus of a resettlement and economic development program called the “Outer Islands Growth Centers Project” (*S18-S20*), largely funded by the Asian Development Bank.

Palmyra atoll is currently inhabited by a small, permanent population of caretakers from The Nature Conservancy and Refuge managers of the US Fish and Wildlife Service, and an itinerant population of visiting scientists (*S21*). The maximum number of people at Palmyra at any given time is 20. Kingman reef has never been permanently inhabited since there is no permanent emergent land.

Population data for the northern Line Islands (Figure S1) since 1921 show that human impact on Palmyra was comparatively isolated and brief, except for the obstruction of circulation within the lagoon, while impacts on Tabuaeran and Kiritimati have been continuous and growing. Populations at Tabuaeran and Kiritimati were similar through 1968. Since that time, Kiritimati has had a consistently higher and faster growing population. The 2005 Census estimates populations of 2,539 at Tabuaeran and 5,115 on Kiritimati. Population data for Kiritimati and Tabuaeran were obtained from Gilbert and Ellice Island Colony and Republic of Kiribati Census reports (*S22-29*).

As an indicator of human impact on the reef, we measured population per km of impacted reef. We used population data from the 2005 Census and the perimeter of impacted reef at the 10 m isobath. Impacted reef was defined as the perimeter of reef adjacent to coastline with known human use based on GIS land-use maps developed by the Ministry of Fisheries & Marine Resources Development, Ministry of Environment, Lands & Agriculture Development, Kiribati Housing Corporation, Telecom Services Kiribati Limited, Ministry of Finance and Economic Planning, Ministry of Public Works Utilities, and the Ministry of Internal & Social Affairs as part of an EU-funded Island System Management project (*S30*). The coastline with human use was measured as the continuous perimeter across the range of village polygons and known fishing grounds. This analysis yielded an estimate of 40.8 km, or 78%, of impacted reef on Tabuaeran versus 47.0 km, or 32% of impacted reef on Kiritimati. The population density along the inhabited coastline of each island is 62.2 people/km reef on Tabuaeran and 108.9 people/km reef on Kiritimati.

Fish Catch and Consumption

With very limited land resources, fishing is critical for subsistence and is one of two primary industries in Kiribati (*S17-S18*, *S31*-*S33*). In particular, Kiritimati is at the center of the country’s strategy for increasing self-reliance and economic growth through the development of subsistence and commercial fisheries.

The Republic of Kiribati has one of the highest rates of per capita fish consumption in the world, estimated at 72-207 kg/person/year (*S33-34*). Based on an average consumption of 140 kg/year and the 2005 Census data, 350 tons/year of fish are consumed in Tabuaeran, and 716 tons/year in Kiritimati. Most of the fish consumed is caught locally.

It is estimated that 96% of housesholds participate in fishing at Kiritimati (*S35*), and 89% at Tabuaeran (*S36*). However, the fishing sectors on each island are very different. Kiritimati has a commercial fishing industry that exports both for consumption and for the aquarium market. Tabuaeran has only semi-commercial fishing with sales between households; 28% of households participate in this activity *(S30)*. The commercial fishing industry on Kiritimati consists of one of the two government-run fishing companies in the country, two private fishing vessels, and eight aquarium fish operators (*S37*-*S39*). Almost 20% of the households in Kiritimati participate in this industry by selling all or part of their catch, while 30% participate in semi-commercial fishing with sales between households (*S35*). The remainder of the fishing activity is for subsistence. Comparing estimates for total annual catch of reef fish during two years where data is available for all types of fishing, the catch on Kiritimati was 435 tons/yr in 2002 versus only 20 tons/yr on Tabuaeran in 2000. The dramatic differences in these annual catch estimates can be attributed in part to the fact that catch from the fore reef is proportionally lower in Tabuaeran and that annual artisanal (subsistence and semi-commercial) reef catch was estimated from surveys of only one week of fishing activity.

The commercial fishing industry began on Kiritimati with the establishment of Kiritimati Marine Exports Limited (formerly The Marine Exports Division) in 1979, which came under the management of the Central Pacific Producers Limited (CPPL) in 2001. Air service and a major CPPL facility with ice plants, freezers, and processing area allow for commercial export on Kiritimati. At the start of the industry, annual exports were between 75 and 97 tons/yr (*S39* cited in *S40*) (Fig. S2). More recently, exports have dropped precipitously from almost 67 tons/yr in 2001 to 11 tons/yr in 2005 (*S41*). A significant portion of the exported fish is reef fish, ranging from 13% to 32% of the total landings from 2001 to 2005. Grouper, snapper, and parrotfish are reported in the top five exported species by weight (*S43*).

Fishermen exporting to the aquarium fish market catch exclusively reef fish (*S37*, *S43* cited in *S42*, *S44*). The aquarium fish industry began on Kiritimati in the early 1980s with one company (*S45*) and now has expanded to eight companies (*S37*). The rapid expansion of this industry is likely due to the high value of aquarium fish and a recent increase in air service to include service to both Fiji and Honolulu. In 2002, the aquarium fish trade on Kiritimati was worth AUD$2,042,667 as compared to AUD$8,272 for all other fish exports (*S46*). Aquarium fish export from 1994 to 2005 has ranged from 27,328 fish to 161,436 fish per year (*S44*). The aquarium fish export from 1994 to 2005 was calculated in terms of biomass (Figure S2) using data on numbers of individual fishes caught by family in 2005 and the average individual biomass of species in these families. There has been a general increase in export over the last ten years, most likely due to an increase in effort. In 2003, 23 tons of fish were exported. In 2005, despite three months without shipments due to a lack of air cargo space, 16 tons of fish were exported. The overwhelming majority of fish exported in 2005 were Pomacanthidae (angelfishes) (91%) (Table S1). Acanthuridae (surgeonfishes) represented only 5% in 2005, whereas other families of fish represented less than 1% each. Some fish more typically thought of as food fish, in the families Serranidae and Lutjanidae, are also exported for the aquarium fish trade.

Compared to commercial fishing, subsistence fishing is the most prevalent fishing activity at both Kiritimati and Tabuaeran. Over 72% of households at Tabuaeran strictly fish for subsistence, compared to 51% of households at Kiritimati (*S35-S37*). Artisanal fishing survey estimates of subsistence reef fish catch at Tabuaeran and Kiritimati are 0.4 tons/week and 8 tons/week, respectively (*S35-S36*). Top predators represent a larger portion of the reef fish catch at Tabuaeran (Lutjanidae = 14%, Elasmobranchs = 15%) as compared to Kiritimati (Lutjanidae = 17%, Elasmobranchs = 5%) (*S35-S36*). In contrast, secondary predators represent a smaller portion of the reef fish catch at Tabuaeran (Serranidae = 14%) than at Kiritimati (Serranidae = 37%). The catch per unit effort (CPUE) for reef fish estimated from one week is roughly equivalent at each island (CPUE = 1.4 kg/hr on Kiritimati, and CPUE= 1.6 kg/hr on Tabuaeran); however, this estimate does not account for differences in gear.

There was some illegal and unreported fishing of sharks and pelagic fishes around Palmyra as recently as the late 1990s, but currently Palmyra Atoll is a U.S. National Wildlife Refuge where fishing is prohibited (*S21*, *S47*). Only a small-scale catch-and-release bonefishing operation is allowed in the lagoon. Kingman Reef is also a U.S. National Wildlife Refuge where fishing is not allowed (*S47*); there are no reported fishing activities at Kingman, although these are difficult to verify because of the extreme isolation of the atoll.

Waste and Sanitation

Less than half of the population in the Republic of Kiribati has access to safe drinking water and sanitation (*S46*). Water contamination by human and other solid waste is a common occurrence in Kiribati because of a lack of toilets and sanitation and waste management infrastructure (*S48*).

In the early part of the 20^th^ century, the northern Line Islands were occupied by seasonal workers for the coconut plantation living in temporary villages. Sanitation methods at the time involved walking to the beach or away from residences for defecation (*S49*). Villages became more permanent around the time when the Republic of Kiribati gained its independence (1979) and some pour-flush toilets and septic tanks were installed (*S49*). Poor maintenance of the tanks and their placement near freshwater lenses led to contamination of drinking water (*S49*). The 2005 Population Census and Household Survey showed that 53% of households on Kiritimati and 20% on Tabuaeran have access to improved sanitation (*S19*). (Improved sanitation refers to a flush toilet or water sealed latrine; whereas, unimproved sanitation refers to attolette, lagoon beach, ocean beach, or public toilet). The number of households with improved sanitation on Kiritimati has increased since 1990 due to recent development projects (*S50-S51*). However, the use of the beach remains common. In the local language, the act of defecating translates to “going to the water” (*S49*). In 2005, 90 households on Kiritimati and 234 on Tabuaeran reported the beach as their primary location for sanitation (*S20*).

At Kiritimati, “pollution and other anthropogenic impacts to reefs in northern (Wilkes) lagoon near major settlements (London, Tabakea, Banana)” were cited as a major problem in community consultations in 1997 (*S42*). Despite development projects, waste and sanitation systems in Kiritimati are either non-existent or in some state of disrepair. Septic tanks are used by the majority of households in the main village of London and to a lesser extent in other areas (*S51*). Also, most septic tanks are full and overflowing because they are being used by multiple households that range in size from 8-33 people (*S51*) and there is no desludging facility (*S52*). Effluent from full septic tanks is more likely to have higher concentrations of disease organisms because the solid waste does not settle out (*S52*). Households may request that their septic tank be emptied by the Water Unit. Emptying of septic tanks is done manually with buckets and the sludge is dumped in the ocean between Tabakea and Main Camp (*S52*).

Prompted by the results of a 1980 AusAID study of water and sanitation on Kiritimati, the Kiritimati Water Supply and Sanitation Project (KWASP) began in 1997. The primary aim for sanitation has been the installation of composting toilets in place of pour-toilets with septic tanks or no toilets (*S49*). Pilot projects were done in 1994; however, by the start of the KWASP project none of the toilets were functioning (*S52*). In 2000, the KWASP project installed 150 composting toilets based on the lessons from the pilot program. By 2002, all the composting toilets were in a state of disrepair and now only a few are being used.

Solid waste is the other main source of pollution in Kiritimati, along with human waste. “Pollution of the lagoon affecting wildlife and the food chain” was cited as a main problem associated with the poor solid waste disposal system (*S52*). In 2000 and 2001, four solid waste cells were set-up by KWASP in Tabakea, Main Camp, Banana (hazardous waste only), and Poland. The Kiritimati Urban Council, which was created in 2004, was placed in charge of managing the cells and running the collection system. The cells are now mismanaged or not managed at all, in part because the employees of the council have no formal training in waste management and none of the waste management manuals that were developed under KWASP were given to the council (*S52*).

Fully functioning waste and sanitation systems are lacking from Tabuaeran and Kiritimati. The beach remains a primary sanitation facility, even on Kiritimati, and there is no functioning system for household waste (*S53*). However, water resource management and waste and sanitation projects, especially on Kiritimati, continue to receive a significant amount of funding and technical support by the Asian Development Bank and other aid agencies (*S50*).

The rules and regulations of the Palmyra Atoll National Wildlife Refuge prompted the development of a state-of-the-art system for waste management, which implies that virtually no polluted water and no solid waste go into the lagoon (*S21*). Kingman Reef has no runoff or local human-derived pollution (*S47*). However, solid waste including plastics, synthetic materials, and glass that originate far away (from the U.S. to Asia), are commonly found on the beaches of Palmyra and the intermittent shoals at Kingman Reef.
