# Supplementary material for: Baselines and Degradation of Coral Reefs in the Northern Line Islands
Source: PLoS One. 2008 Feb 27;3(2):e1548. doi: 10.1371/journal.pone.0001548 (PMC2244711; doi:10.1371/journal.pone.0001548)
Supplement: Table S1 — Aquarium reef fish catch for export at Kiritimati (April–December, 2005). (0.03 MB DOC) [file pone.0001548.s002.doc]

**Table S1.** Kiritimati Aquarium Fish Export by Family (April-December, 2005).

| Family | Biomass (kg) | Number of individuals |
| --- | --- | --- |
| Pomacanthidae | 15,565.3 | 94,893 |
| Acanthuridae | 892.3 | 4,776 |
| Serranidae | 147.1 | 596 |
| Pomacentridae | 133.3 | 15,308 |
| Balistidae | 128.2 | 287 |
| Labridae | 79.9 | 608 |
| Chaetodontidae | 71.5 | 424 |
| Tetraodontidae | 60.4 | 334 |
| Lutjanidae | 3.6 | 19 |
| Cirrhitidae | 1.3 | 35 |
| Mullidae | 0.5 | 4 |
| Total | 17,083.4 | 117,284 |
