# Supplementary material for: Baselines and Degradation of Coral Reefs in the Northern Line Islands
Source: PLoS One. 2008 Feb 27;3(2):e1548. doi: 10.1371/journal.pone.0001548 (PMC2244711; doi:10.1371/journal.pone.0001548)
Supplement: Table S2 — Historical accounts of shark abundance in the Line Islands. (0.05 MB DOC) [file pone.0001548.s003.doc]

**Table S2.** Historical accounts of shark abundance in the northern Line Islands, Republic of Kiribati.

| **Year** | **Location** | **Description** |
| --- | --- | --- |
| 1777 | Kiritimati [S54] | "On every side of us swam Sharks innumerable, & so voracious that they bit our oars & rudder, & I actually struck my hanger 2 inches into the back of one whilst he has the rudder between his teeth. The boats fishing for Cavallies & etc. in Shallow water, carried long pikes to keep the Sharks from the Bait. On board the Ships, the Sailors caught great numbers, & as these two beings are constantly at war with each other, contrived a hundred ways to torment them....” |
| 1777 | Kiritimati [S54] | "...the Sharks came about the boats in great Number & carried away many of the fishing Lines." |
| 1838 | Kiritimati [S55] | "It [the lagoon] is also much infested with sharks." |
| 1798 | Tabuaeran [S56] | "The sharks here are very numerous, and while the boat was on her passage into the bay, before she entered the pass, they became so exceedingly ravenous around her, and so voracious withal, as frequently to dart at, and seize upon her rudder and the oars, leaving thereon many marks of their sharp teeth and powerful jaws; but so soon as she left the pass and entered the bay, they deserted her, their stations being instantly occupied by multitudes of fish, less rapacious, yet infinitely more valuable." |
| 1893 | Tabuaeran [S57] | "Sharks at Fanning were numerous but not large, as vicious as the devil, as supple as whalebone, and the darkest I've ever seen. Once Jimmy Greig took me to see droves of them entering the lagoon by a shallow passage at high spring tide, slowly feeling their way in two or three feet of water. When we foolishly crossed the inlet Jimmy was bitten on the heel, not seriously, however, through the wound bled profusely. Attracted by the blood, a shoal of ravenous monsters rushed for us, and we rushed ashore. Only a miracle could have saved us if we had stumbled and fallen." |
| 1837 | Palmyra [S58] | "Sharks were numerous, and so voracious that they attacked both the boat and the steering oar as we were pulling ashore." |
| 1893 | Palmyra [S57] | "Our other capture was a huge shark, the largest we caught on the entire cruise, twenty-two feet and eight inches in length by eight and one half feet in girth amidships. When dried the fins and tail weighed twenty-five pounds; the liver yielded fourteen gallons of excellent oil, enough to fill the last of our containers. In its stomach was the body of a shark we had killed the day before, itself no less than eight feet long." |
| 1937 | Palmyra [S59] | "There is a beautiful lagoon in the center of the islands and it's filthy with sharks and fish.” |
