# Supplementary material for: Baselines and Degradation of Coral Reefs in the Northern Line Islands
Source: PLoS One. 2008 Feb 27;3(2):e1548. doi: 10.1371/journal.pone.0001548 (PMC2244711; doi:10.1371/journal.pone.0001548)
Supplement: Table S3 — Non-coral invertebrate abundance on the northern Line Islands. (0.12 MB DOC) [file pone.0001548.s004.doc]

**Table S3.** Non-coral macroinvertebrate abundance in the northern Line Islands. Data are means (number of individuals 100 m^-2^) and standard deviation (SD), for the most abundant species and taxonomic groups. N=number of sites per island.

|  |  | Kingman | Palmyra | Tabuaeran | Kiritimati |
| --- | --- | --- | --- | --- | --- |
|  |  | N=10 | N=10 | N=8 | N=11 |
| CEPHALOPODS |  |  |  |  |  |
| *Octopus cyanea* | Means | 0.00 | 0.00 | 0.00 | 0.53 |
|  | SD | 0.00 | 0.00 | 0.00 | 0.56 |
| GASTROPODS |  |  |  |  |  |
| *Turbo argyrostomus* | Means | 0.42 | 0.00 | 0.00 | 0.08 |
|  | SD | 1.06 | 0.00 | 0.00 | 0.25 |
| BIVALVES |  |  |  |  |  |
| *Tridacna maxima* | Means | 0.75 | 0.08 | 0.00 | 0.45 |
|  | SD | 0.83 | 0.26 | 0.00 | 0.78 |
| ECHINOIDS | Means | 24.58 | 0.00 | 1.46 | 8.18 |
|  | SD | 20.92 | 0.00 | 4.12 | 26.86 |
| *Echinotrix diadema* | Means | 24.50 | 0.00 | 0.00 | 0.00 |
|  | SD | 20.94 | 0.00 | 0.00 | 0.00 |
| *Diadema paucispinum* | Means | 0.00 | 0.00 | 0.00 | 8.21 |
|  | SD | 0.00 | 0.00 | 0.00 | 26.88 |
| *Echinometra mathaei* | Means | 0.00 | 0.00 | 1.46 | 0.08 |
|  | SD | 0.00 | 0.00 | 4.12 | 0.25 |
| *Echinostrephus aciculatus* | Means | 0.08 | 0.00 | 0.00 | 0.00 |
|  | SD | 0.26 | 0.00 | 0.00 | 0.00 |
| HOLOTHURIANS | Means | 1.08 | 0.08 | 0.00 | 0.76 |
|  | SD | 1.62 | 0.26 | 0.00 | 1.08 |
| *Synapta maculata* | Means | 0.00 | 0.00 | 0.00 | 0.15 |
|  | SD | 0.00 | 0.00 | 0.00 | 0.34 |
| *Actinopyga mauritiana* | Means | 0.25 | 0.00 | 0.00 | 0.00 |
|  | SD | 0.40 | 0.00 | 0.00 | 0.00 |
| *Pearsonothuria graeffei* | Means | 0.00 | 0.08 | 0.00 | 0.00 |
|  | SD | 0.00 | 0.26 | 0.00 | 0.00 |
| *Bohadschia argus* | Means | 0.00 | 0.00 | 0.00 | 0.53 |
|  | SD | 0.00 | 0.00 | 0.00 | 0.67 |
| *Bohadschia marmorata* | Means | 0.00 | 0.00 | 0.00 | 0.08 |
|  | SD | 0.00 | 0.00 | 0.00 | 0.25 |
| *Holothuria whitmaei* | Means | 0.17 | 0.00 | 0.00 | 0.00 |
|  | SD | 0.35 | 0.00 | 0.00 | 0.00 |
| *Holothuria atra* | Means | 0.67 | 0.00 | 0.00 | 0.00 |
|  | SD | 1.35 | 0.00 | 0.00 | 0.00 |
| ASTEROIDS | Means | 0.17 | 0.00 | 0.00 | 0.45 |
|  | SD | 0.53 | 0.00 | 0.00 | 1.08 |
| *Culcita novaeguineae* | Means | 0.08 | 0.00 | 0.00 | 0.00 |
|  | SD | 0.26 | 0.00 | 0.00 | 0.00 |
| *Linckia laevigata* | Means | 0.00 | 0.00 | 0.00 | 0.45 |
|  | SD | 0.00 | 0.00 | 0.00 | 1.08 |
| *Fromia milleporella* | Means | 0.08 | 0.00 | 0.00 | 0.00 |
|  | SD | 0.26 | 0.00 | 0.00 | 0.00 |
| ANTHOZOANS | Means | 5.50 | 6.40 | 3.00 | 5.78 |
|  | SD | 0.71 | 2.95 | 2.62 | 2.54 |
| PORIFERANS | Means | 0.00 | 0.30 | 0.50 | 0.56 |
|  | SD | 0.00 | 0.67 | 0.76 | 0.53 |
