# Supplementary material for: Baselines and Degradation of Coral Reefs in the Northern Line Islands
Source: PLoS One. 2008 Feb 27;3(2):e1548. doi: 10.1371/journal.pone.0001548 (PMC2244711; doi:10.1371/journal.pone.0001548)
Supplement: Table S4 — Non-coral invertebrate data analysis: Median test for comparison of species abundance between atolls. (0.05 MB DOC) [file pone.0001548.s005.doc]

**Table S4.** Non-Coral Invertebrate Data Analysis. Median test for comparison of species abundance between atolls. df=3. Atoll differences were calculated using a multiple post-hoc comparison; differences are significant with p<0.05.

| TAXON | Chi-square | p | Atoll differences |
| --- | --- | --- | --- |
| CEPHALOPODS |  |  |  |
| *Octopus cyanea* | 18.05 | 0.0004 | KIR>(TAB=PAL=KIN=0) |
| GASTROPODS | |  |  |
| *Turbo argyrostomus* | 3.66 | 0.30 |  |
| BIVALVES |  |  |  |
| *Tridacna maxima* | 10.13 | 0.018 | KIN>KIR>PAL>(TAB=0) |
| ECHINOIDS | 27.7 | <.0001 | KIN>KIR>TAB>(PAL=0) |
| *Echinotrix diadema* | 39 | <.0001 | KIN>(TAB=PAL=KIR=0) |
| *Diadema paucispinum* | 2.61 | 0.46 |  |
| *Echinometra mathaei* | 2.33 | 0.51 |  |
| *Echinostrephus aciculatus* | 2.98 | 0.40 |  |
| HOLOTHURIANS | 8.74 | 0.03 | KIN>KIR>PAL>(TAB=0) |
| *Synapta maculata* | 5.37 | 0.15 |  |
| *Actinopyga mauritiana* | 9.42 | 0.02 |  |
| *Pearsonothuria graeffei* | 2.98 | 0.40 |  |
| *Bohadschia argus* | 14.6 | 0.002 |  |
| *Bohadschia marmorata* | 2.61 | 0.46 |  |
| *Holothuria whitmaei* | 6.11 | 0.11 |  |
| *Holothuria atra* | 9.42 | 0.02 |  |
| ASTEROIDS | 3.28 | 0.35 |  |
| *Culcita novaeguineae* | 2.98 | 0.40 |  |
| *Linckia laevigata* | 5.37 | 0.15 |  |
| *Fromia milleporella* | 2.98 | 0.40 |  |
| ANTHOZOANS | 3.86 | 0.28 |  |
| PORIFERANS | 3.78 | 0.29 |  |
