# Supplementary figures and images for: Baselines and Degradation of Coral Reefs in the Northern Line Islands
Source: PLoS One. 2008 Feb 27;3(2):e1548. doi: 10.1371/journal.pone.0001548 (PMC2244711; doi:10.1371/journal.pone.0001548)

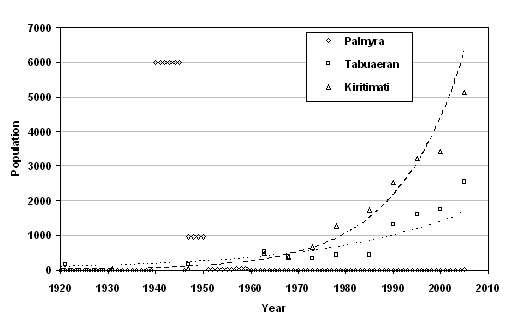

Supplement: Figure S1 — Population data for the northern Line Islands (0.05 MB TIF) [file pone.0001548.s006.tif]

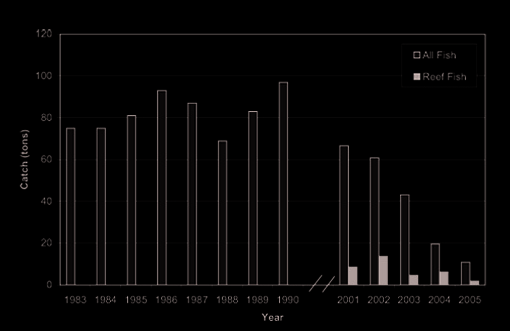

Supplement: Figure S2 — Total annual reef fish catch at Kiritimati (0.68 MB TIF) [file pone.0001548.s007.tif]

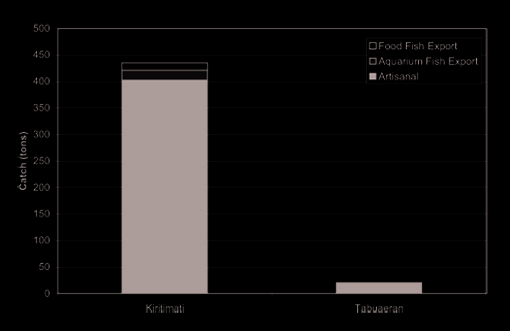

Supplement: Figure S3 — Annual Fish Catch for Export on Kiritimati (0.66 MB TIF) [file pone.0001548.s008.tif]

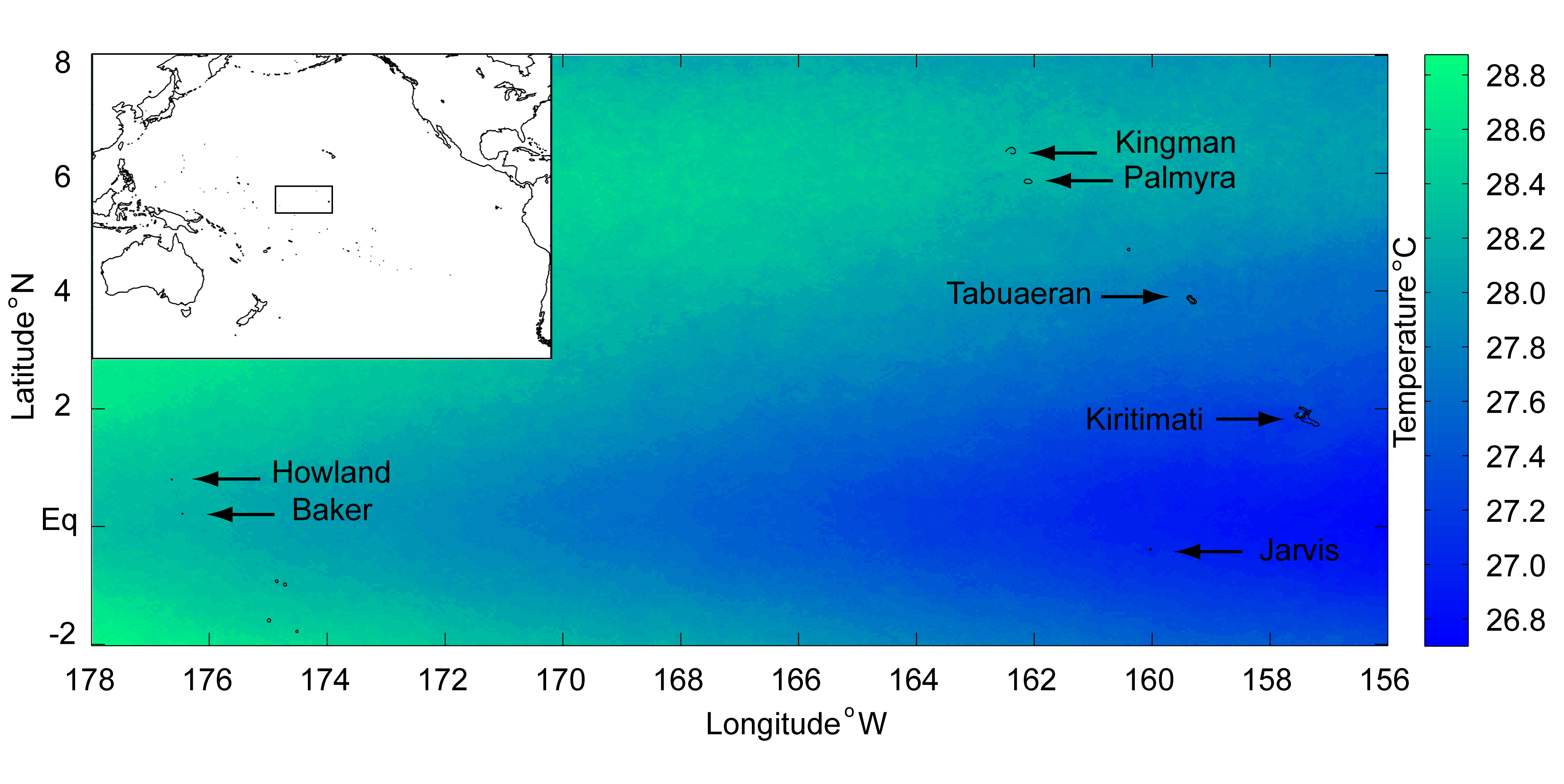

Supplement: Figure S4 — Map of the central Pacific, including atolls of the Line and Phoenix Islands. Colors reflect mean sea surface temperatures for August 2005. Note the latitudinal gradient of temperature determined by meeting of the equatorial current and countercurrent (3.43 MB TIF) [file pone.0001548.s009.tif]
